# Exploring User Perspectives on Brief Reflective Questioning Activities for Stress Management: Mixed Methods Study

**DOI:** 10.2196/47360

**Published:** 2024-02-08

**Authors:** Ananya Bhattacharjee, Pan Chen, Abhijoy Mandal, Anne Hsu, Katie O'Leary, Alex Mariakakis, Joseph Jay Williams

**Affiliations:** 1 Department of Computer Science University of Toronto Toronto, ON Canada; 2 School of Electronic Engineering and Computer Science Queen Mary University London United Kingdom; 3 Google Seattle, WA United States

**Keywords:** reflection, mental health, stress, reflective questioning activity, RQA, brief intervention, computer-mediated communication, email, SMS text messaging, mobile phone

## Abstract

**Background:**

Current online interventions dedicated to assisting individuals in managing stress and negative emotions often necessitate substantial time commitments. This can be burdensome for users, leading to high dropout rates and reducing the effectiveness of these interventions. This highlights an urgent need for concise digital activities that individuals can swiftly access during instances of negative emotions or stress in their daily lives.

**Objective:**

The primary aim of this study was to investigate the viability of using a brief digital exercise, specifically a reflective questioning activity (RQA), to help people reflect on their thoughts and emotions about a troubling situation. The RQA is designed to be quick, applicable to the general public, and scalable without requiring a significant support structure.

**Methods:**

We conducted 3 simultaneous studies. In the first study, we recruited 48 participants who completed the RQA and provided qualitative feedback on its design through surveys and semistructured interviews. In the second study, which involved 215 participants from Amazon Mechanical Turk, we used a between-participants design to compare the RQA with a single-question activity. Our hypotheses posited that the RQA would yield greater immediate stress relief and higher perceived utility, while not significantly altering the perception of time commitment. To assess these, we measured survey completion times and gathered multiple self-reported scores. In the third study, we assessed the RQA’s real-world impact as a periodic intervention, exploring engagement via platforms such as email and SMS text messaging, complemented by follow-up interviews with participants.

**Results:**

In our first study, participants appreciated the RQA for facilitating structured reflection, enabling expression through writing, and promoting problem-solving. However, some of the participants experienced confusion and frustration, particularly when they were unable to find solutions or alternative perspectives on their thoughts. In the second study, the RQA condition resulted in significantly higher ratings (*P*=.003) for the utility of the activity and a statistically significant decrease (*P*<.001) in perceived stress rating compared with the single-question activity. Although the RQA required significantly more time to be completed (*P*<.001), there was no statistically significant difference in participants’ subjective perceived time commitment (*P*=.37). Deploying the RQA over 2 weeks in the third study identified some potential challenges to consider for such activities, such as the monotony of doing the same activity several times, the limited affordances of mobile phones, and the importance of having the prompts align with the occurrence of new troubling situations.

**Conclusions:**

This paper describes the design and evaluation of a brief online self-reflection activity based on cognitive behavioral therapy principles. Our findings can inform practitioners and researchers in the design and exploration of formats for brief interventions to help people with everyday struggles.

## Introduction

### Background

Computer-mediated communication (CMC) platforms offer accessible resources to assist people in managing their stress and negative emotions [1,2]. Nevertheless, current online interventions can be time-consuming and inconvenient [3], necessitating users to commit to a series of hour-long sessions to achieve optimal results. Social media groups and SMS text messaging programs also require a substantial time commitment from users to deliver maximum benefit [3,4]. Although research demonstrates that these programs can be as effective as in-person therapy [5,6], the considerable time investment required may lead to high dropout rates. Consequently, the convenience of online resources is paramount in enhancing their efficacy and user engagement [7].

Therefore, we investigate whether it might be possible to construct a brief digital activity (as simple as answering questions in a web form) that people can easily reference or practice when they experience negative thoughts and emotions in their daily lives. Through a simple interface with a series of questions, we explore whether a brief reflective questioning activity (RQA) could prompt people to reflect on a stressful situation. This process of articulating thoughts and emotions has the potential to enhance an individual’s understanding of their personal challenges and foster a sense of self-agency [8,9], eventually strengthening their belief in their own ability to manage stress and negative emotions [10]. Brief activities such as RQAs, which require minimal effort and may provide tangible benefits, can also serve as a stepping stone to more extensive treatments [11,12]. This approach has tremendous potential in terms of convenience as well because such RQAs can be delivered to anyone anytime via email, app, and SMS text message. We posit that activities such as this can be made generalizable enough so that they can be adapted to fit the unique needs and preferences of individuals from diverse backgrounds and situations; for example, an individual experiencing stress at work may use reflective questioning to reflect on their thoughts and emotions related to a difficult conversation with a coworker, and another individual may adapt the same activity to reflect on their feelings after a breakup or a family conflict.

In our work, we draw on insights from clinical psychology and human-computer interaction literature on how to design brief RQAs that are helpful for people to manage psychological well-being and adopt healthy behaviors [13-15]. Murgraff et al [15] demonstrated that a persuasive 2-page pamphlet distributed at the beginning of an 8-week study period and informing female university students about recommended drinking limits could effectively reduce unhealthy drinking behaviors. Carney et al [16] used a similar intervention to support adolescent users of substances and their caregivers. These studies suggest that extensive interventions are not always necessary to foster healthy behavior; providing a brief guideline with crucial information and actionable practices for self-directed application can be beneficial too.

Our work is focused on the goal of promoting self-reflection, a crucial component of cognitive behavioral therapy (CBT) [17] and psychology in general. One can understand self-reflection as a person’s conscious effort to understand and reevaluate their own thoughts regarding any situations, thoughts, or feelings [18,19]. Self-reflection is often the driving force that converts one’s intentions into action [20]. Furthermore, it allows an individual to view situations from a different perspective, enabling them to understand the opinions of others [20,21]. In recent years, researchers have incorporated many reflective activities into mental health and behavior change interventions, particularly through mobile phone apps that show users summaries of their mood or physical activity [22-24]. Other digital tools have attempted to promote self-reflection through conversational agents [25,26]. As evident with the recent emergence of chatbots such as ChatGPT [27,28], conversational agents continue to become more sophisticated in parallel with advances in natural language processing, but they are still limited in their ability to have nuanced and empathetic conversations [27]. Furthermore, the literature suggests that back-and-forth conversations are not always necessary to elicit self-reflection because asking probing questions with the words *why* or *how* can be enough to increase one’s own understanding of a problem [29,30].

However, there are several reasons to speculate that brief RQAs may not effectively help individuals manage their stress. First, prompts for self-reflection may not provide people with something concrete or tangible (eg, new information or social validation) and might require repeated exposure to yield benefits that people can see [31,32]. Moreover, it is unclear whether people would see value in answering reflective questions and whether an extended series of questions would add much value. Answering a static set of questions could not only be perceived as a waste of people’s time but also surface more negative emotions without a conversational partner to give input. Furthermore, people might prefer knowing that their thoughts and emotions are being shared with another person rather than relying on themselves to gain benefits.

Drawing upon these potential opportunities and challenges, we set the following guiding principles for our exploration:

- Minimal time commitment: the activity should be simple enough so that people can complete it in 15 minutes—the equivalent of a midday coffee break at work or a fraction of a person’s morning routine.
- Applicability to the general public: the activity should not be targeted toward a particular domain, culture, or population. In other words, the activity should be generalizable to the point where people can adapt it to their own context and situation.
- Scalability: the activity should be implemented and deployed in a way that does not need a significant support structure. This means that the activity should not require a live conversational partner or intensive scaffolding (eg, tutorial videos).

To investigate the feasibility, challenges, and opportunities in the design of digital RQAs, we created a design probe that asks people to answer a series of 9 questions to reflect on a troubling situation. The questions in our RQA are intended to help people articulate their thoughts and emotions about the situation using principles from CBT [33]. We leveraged thought records [34] and behavioral chaining analysis [10], which are techniques that encourage people to connect their thoughts, experiences, and emotions to identify triggers that generate negative patterns and come up with alternative ways of thinking.

We provide insights into the design of our RQA and how it was experienced by users, which we hope will inform the design of future interventions with similar goals. We gathered these observations through 3 studies. For our first study, we used a convenience sample of crowdworkers and university students to administer the RQA and obtain qualitative feedback on the design of the activity. In our second study, we investigated whether the perceived benefits of going through an RQA outweighed the additional time commitment required to answer a series of probing questions. In our third and final study, we investigated the potential impact of the RQA when delivered repeatedly over a 2-week period in a real-world context; we also explored the implications of distributing the RQA over email versus SMS text message. The design of our RQA was kept constant across all 3 studies so that we could maintain consistency across evaluations and determine which observations held true across the different scenarios.

We found that the structured analysis supported by our RQA helped people reduce their stress and identify solutions for improvement. Although our RQA consisted of 9 questions, people did not complain about the time commitment required to complete it and generally wrote thoughtful responses to the prompts. However, deploying the RQA over the course of 2 weeks raised some potential challenges, including the monotony of doing the same activity several times, the limited affordances of mobile phones, and the importance of having the prompts align with the occurrence of new troubling situations. These highlight design considerations and opportunities for researchers and practitioners to consider as they develop their own digital RQAs, such as giving users control over the frequency of prompts and automated question personalization.

### Main Contribution

In summary, our main contribution is an investigation into whether people see value in a brief digital RQA without a conversational partner for interaction or advice. We deliver this contribution in four parts: (1) the creation of an RQA probe that people can complete on their computer or mobile phone to reflect upon a stressful situation; (2) insights into the value and pitfalls of RQAs gathered via surveys completed by, and interviews with, 42 Amazon Mechanical Turk (AMT) participants and 6 university students; (3) evidence that people see value in an RQA compared with a baseline activity via a comparison study run on AMT with 215 participants; and (4) observations and design considerations from a 2-week deployment of our RQA using different CMC platforms.

## Methods

### Overview

In this section, we first discuss the design of our RQA and then describe the logistics of the 3 studies we conducted. The studies were conducted simultaneously with the same RQA design to explore different aspects of the intervention. Study 1 involved gathering feedback on the qualities of the RQA from a broad demographic using surveys and semistructured interviews. In study 2, the perceived benefits of the RQA were compared with those of a shorter baseline activity with the goal of determining whether the additional time commitment required to complete the RQA was justified by the benefits of the intervention. Study 3 aimed to explore how people would perceive the RQA during their everyday lives and how best to prompt engagement using email and SMS text messaging.

### The Design of Our RQA

Our research team, which consists of graduate students and faculty members with experience in psychology and human-computer interaction, was guided by existing CBT resources to create an RQA that helps people reflect on a troubling situation in their lives. We first reviewed popular CBT apps and websites intended for personally guided use (eg, Youper [35], Depression CBT Self-Help Guide [36], KokoBot [37], and Woebot [38]) to identify the techniques they used to provide benefits to users. In particular, we found that these resources leverage several components of a CBT exercise called a thought record [10]. A thought record is a worksheet with a grid that includes 5 columns: situation, thoughts, emotions, behaviors, and alternative thoughts. The exercise aims to encourage behavioral chaining—a process through which people draw connections between their thoughts and emotions to identify triggers and irrational thoughts—revealing potential opportunities to reframe their way of thinking [10,39].

Researchers have identified several benefits to thought records and behavioral chaining. Thought records can help people recall memories of prior events that were initially assumed to be unimportant [40]. Identifying the full timeline of an event can help people recognize their own faulty behavior patterns, thus preparing them for similar events in the future [41]. Moreover, informal exposure to negative experiences can increase one’s ability to tolerate troubling situations [42] or recover from problematic behaviors (eg, binge drinking and self-harming) [10]. Thought records are typically introduced as CBT homework assignments that patients can complete between visits with a trained professional, providing them with the scaffolding to complete the activity on their own.

Our RQA attempts to distill this exercise into a brief guided activity that can be completed on a person’s computer or mobile phone without the need for external support. After writing a collection of brief questions to encapsulate these concepts, we iteratively added, removed, revised, and reordered the questions until we reached the RQA structure shown in Table 1. Our primary design goal was to give people a structured activity they could use independently to organize their thoughts. Inspired by thought records and behavioral chaining, our activity guides users through the following line of thinking: trigger → thought → feeling → behavior [9,10].

We first start by asking the user to think about a stressful situation and write about it in as much detail as they like. Prior work suggests that this sort of open-ended question allows users to open up about their problems and be comfortable with the activity [9]. The next 5 questions (Q2-Q6) become more specific, asking users to identify the most important stressor, the most troubling thoughts and feelings, and the behaviors that come from these thoughts and feelings. The seventh question then asks users to retype the details of the situation in a structured format. Beyond leading people through the process of behavioral chaining, these questions allow users to iterate upon their initial thoughts regarding their stressful situation. The structured format in the seventh question is also designed to help users draw connections between several components of their situation. This leads to the eighth question, which challenges the user’s mental process by asking them whether they believe that the trigger justifies their thoughts. Doing so can help people identify flaws in their logic or possible cognitive distortions [10]. The final question asks the user to explore alternative ways of thinking that would enable them to see the problem from a different perspective and induce a different emotion [43].

We presented our RQA to 4 clinical psychologists with expertise in CBT to validate its construction and help us consider the best ways of evaluating it. The psychologists verified that our RQA is aligned with activities that would be used in psychotherapy, but they also remarked that the questions focused on advanced techniques that were usually introduced only after several sessions of evaluation and psychoeducation. They suspected too that people might find the activity too lengthy or that people might not know how to respond to some of the questions; 1 psychologist even posited that >2 questions might be excessive for an online format without a conversational partner. The study that follows in this paper demonstrates that although these concerns were warranted, participants found value in the additional line of questioning.

**Table 1:** The questions that compose our reflective question activity. The design of these questions is influenced by thought records and behavioral chaining. Before seeing these questions, participants were provided with the following prompt: “Think of a particular situation where you felt stressed or had a negative emotion, which you can try to reflect on as you go through this activity. It could be a current situation, one in the past, or one you anticipate in the future.”

Questions	Example response	Purpose
“Q1. What’s the situation? Feel free to explain it in as much detail as you’d like.”	“My son has moved away and left no way for me to get in contact with him.”	Provides context for the activity
“Q2. What part of the situation is the most troubling?”	“The fact that he does not care enough to reach out to me and let me know he is safe.”	Sets an agenda for the rest of the activity
“Q3. What are you thinking to yourself?”	“I hope he is okay and safe. I wonder why he would do this. I thought we had a good relationship.”	Identifies troubling thoughts
“Q4. What thought is the most troubling?”	“I don’t know if he is safe.”	Focuses attention on the most troubling thought
“Q5. What do you feel when you think this?”	“Panicked and worried.”	Reinforces the core CBT^a^ principle that thoughts trigger feelings
“Q6. When you have these feelings, what actions do you take? What actions do you avoid?”	“I try to refocus my thoughts on something else. I try to avoid thinking about what bad things could be happening to him.”	Identifies behaviors that are caused by the cascading effect of thoughts and feelings
“Q7. Retype the summary of the situation in the following format: “Trigger: “Thought: “Feeling: “Behavior:”	“I am triggered by thoughts of my son taking off and not staying in contact. I think about all the bad things that could happen and why he would do this. I feel panicked and worried. When feeling this way I try to think about other things and not focus on the negative of the situation.”	Synthesizes past reflection by highlighting the connection between the trigger and its manifestations
“Q8. Consider whether the trigger truly justifies this type of thinking. Explain below.”	“The trigger does justify it. This is my child that I raised. I no longer know where he is, I cannot get in touch with him and I don’t know if he is okay.”	Challenges potentially negative thought patterns
“Q9. If you were to explore an alternative line of thinking, how would you do it?”	“I raised my child to be independent and he is trying to exercise that independence for the first time in his life. He needs me to take a step back for a while so that he can do this on his own.”	Encourages alternative thoughts that can provoke different feelings and behaviors

^a^CBT: cognitive behavioral therapy.

### Study 1: User Perspectives After Onetime Use of Our RQA

#### Overview

Our first study gathered qualitative feedback on the qualities that people saw in the proposed RQA irrespective of other factors (eg, when it was being used and how it compared with other interventions). We used surveys to collect diverse feedback from a broad demographic. Subsequently, we used semistructured interviews to gather deeper insights into some of the salient topics.

#### Participants

We initially recruited 50 participants from AMT. Participants were required to have a minimum approval rating of 95%. We did not incorporate explicit attention check questions in our surveys but implemented a thorough manual review process to ensure data quality. Two independent members of our research team examined each response, discarding any that were incomplete or contained nonsensical or irrelevant content. Because of data quality issues, we discarded data from 8 (16%) of the 50 participants, leaving us with a final sample of 42 (84%). This cohort of 42 AMT crowdworkers included 35 (83%) men and 7 (17%) women, with an average age of 34.6 (SD 9.99) years. We identified these participants as M1 to M42, and they were compensated CAD $4 (US $2.97) for their time. We also recruited 6 additional people via email and word of mouth from a university campus community to serve as interview participants. This cohort of 6 people included 1 (17%) man and 5 (83%) women with an average age of 19.7 years. We identified these participants as L1 to L6, and they were compensated CAD $15 (US $11.15) for their time. There were no inclusion criteria because we were interested in observing how our RQA would be perceived by the general population.

#### Study Procedure

All participants were asked to complete the RQA online on the Qualtrics survey platform (Qualtrics International Inc), with all questions being presented on a single page. The data from this survey were saved and made accessible to the research team. After participants finished the RQA, they were requested to provide their feedback on the activity through a separate survey. The questions included, but were not limited to, the following: “How did this activity affect your stress levels?” “How did you feel about answering these questions in this online format?” and “Was any part of the activity not helpful or could be improved?” The university students also answered similar questions, although they participated in semistructured interviews immediately after completing our RQA. The interviews took 45 to 60 minutes and were held either in person or through different videoconferencing platforms.

#### Data Analysis

The survey responses were analyzed using a thematic analysis approach [44]. After the interviews were transcribed, 2 researchers examined the data together to familiarize themselves with the general sentiments of the participants. The researchers then individually applied the open coding process [45] to a subset of the data to develop their own preliminary codebooks. After sharing their codebooks with one another, the researchers held multiple discussions to consolidate the codes into a shared codebook. Next, they applied this codebook to a different subset of the data and again refined the codebook. Finally, the researchers reached a consensus and applied the final codebook to separate halves of the data.

The interview transcripts were also analyzed using open coding. However, because the interviews aimed to gain deeper insights into what people had to say in the surveys, we used the same codebook generated from the survey responses.

### Study 2: Comparing the RQA With a Baseline

#### Overview

The observed benefits of reflective questioning may be attributed to the act of discussing a troubling situation rather than the structured questions themselves. Furthermore, clinical psychologists raised concerns that asking participants to answer >2 questions may be overwhelming. To investigate these possibilities, we compared the effects of the RQA with those of simply asking participants to discuss their troubling situation without structured questions. In this baseline activity, participants were required to write about a stressful situation in as much detail as possible in response to a single question. If the structured questions in the RQA provided additional benefits over the baseline activity despite the added time commitment, we posited that the RQA would warrant further exploration as a tool for promoting self-reflection.

#### Participants

For study 2, we again used AMT and adhered to the same participant recruitment and data quality assurance procedures from study 1. We initially recruited 255 participants for this study. Of the 255 participants, after the data screening process, we excluded 40 (15.7%; n=17, 43% from the baseline group and n=23, 58% from the RQA group) owing to issues related to data quality. This led to a final participant count of 215, with 111 (51.6%) individuals randomly assigned to the baseline group and 104 (48.4%) to the RQA group. Our study included participants of different genders, with 61.9% (133/215) identifying as men, 35.8% (77/215) identifying as women, and 2.3% (5/215) preferring not to disclose their gender. The mean age of the participants was 33.8 (SD 9.51) years. As with study 1, all participants were compensated CAD $4 (US $2.97) for their participation, and there were no inclusion criteria.

#### Study Procedure

The study had a between-participants design in which participants were randomized into 1 of 2 conditions. The first condition, which we consider the RQA condition, entailed participants completing our 9-question RQA. The second condition, which we call the baseline condition, asked participants to reflect on a troubling situation they were experiencing in as much detail as they wished, answering only a single question.

We expected the RQA to take longer to complete than the baseline condition, given that it involved answering more questions. However, we were also interested in participants’ perceptions of the activity’s length and the value they placed on the additional time spent. By using a between-participants design, the study aimed to assess whether completing the RQA would lead to differences in outcomes compared with the baseline condition.

#### Data Analysis

We collected data before and after participants completed their respective activities to evaluate the hypotheses outlined in the following subsections.

#### Hypothesis 1 (Perceived Benefits)

We hypothesized that participants in the RQA condition would experience more instantaneous stress relief from completing the activity than those in the baseline condition.

To evaluate this hypothesis, we asked participants to rate how useful they felt the activity was. We call this measure *perceived utility*, and it was measured using a 7-point scale (ranging from −3 for *strongly disagree* to +3 for *strongly agree*). We also asked participants to rate the degree to which they were feeling troubled about their selected situation before and after the activity. These ratings were provided using an 11-point scale (ranging from −5 to +5) to increase the resolution with which people could express their stress. We call the difference between the ratings before and after the activity the *perceived stress change*, with positive values indicating a decrease in stress. Both *perceived utility* and *perceived stress change* were compared across conditions using independent samples 1-tailed Welch *t* tests. For each measure, the null hypothesis (H_0_) was that the mean for the RQA condition would be less than, or equal to, the mean for the baseline condition. By contrast, the alternative hypothesis (H_a_) was that the mean for the RQA condition would be greater than the mean for the baseline condition.

#### Hypothesis 2 (Elapsed Time)

We hypothesized that participants in the RQA condition would take more time to complete the activity than those in the baseline condition; yet the perceived time commitment would not be significantly different.

To evaluate this hypothesis, we recorded the time it took for participants to complete the activity, the number of words they typed across all questions, and a self-reported rating using a 7-point scale (ranging from −3 to +3) of whether they felt the activity was worth their time. We call these measures *completion time, response length,* and *perceived time commitment*, respectively. All 3 measures were compared across conditions using independent samples 1-tailed Welch *t* tests. For each measure, the null and alternative hypotheses were set in a similar manner as detailed for hypothesis 1.

### Study 3: Observing Repeated Engagement With the RQA

In our third and final study, we aimed to assess the effectiveness of the RQA in a real-world setting as a periodic intervention and explore the most effective ways to prompt engagement through low-cost asynchronous CMC platforms such as email and SMS text messaging.

#### Participants

We recruited 11 participants (n=8, 73% men and n=3, 27% women) with an average age of 20.6 years. Participants were recruited via email invitations and word of mouth from the same university campus community as study 1 without any inclusion criteria. We refer to these participants as D1 to D11. Participants were not compensated for completing our RQA to avoid undue influence on their level of engagement; however, they were compensated CAD $10 (US $7.43) for completing surveys and CAD $15 (US $11.15) for the interviews.

#### Study Procedure

Participants were recruited to take part in our study for 2 weeks. During the enrollment phase, participants were asked to specify the hours during each day when they would prefer to receive a notification to complete the RQA. They were asked to provide separate preferences for email and SMS text message, and they were allowed to select multiple times during a given day. Participants were then randomized into 1 of 2 groups. One group received emails during the first week and SMS text messages during the second week, whereas the other group experienced the reverse. The notifications prompted participants to complete the RQA and provided them with a link that took them to a web page containing the RQA. We used the same link each time, and participants were aware of this fact.

Participants were prompted to complete the activity once per day for up to 3 days within a given week, similar to what has been done in previous work [46]. Of the 11 participants, 8 (73%) were available for >3 days, and the days on which they received prompts were randomly selected, whereas 3 (27%) were available for <3 days (D2, D8, and D9), and they received a message on every day of their availability.

At the end of the study, participants were asked to complete an exit survey containing questions about their overall experience and their CMC preferences in the context of the RQA. They were then invited to a semistructured interview session to elaborate on their experience. The interviews lasted 15 to 30 minutes, with frequent topics including the barriers people faced while completing the RQA, the applicability of the RQA to their lives, and the trade-offs of being prompted to complete the RQA repeatedly. Of the 11 participants, 7 (64%) took part in the interviews. The interviews were conducted over the Zoom teleconferencing platform (Zoom Video Communications, Inc).

#### Data Analysis

We recorded a variety of data to assess how people engaged with our RQA. We measured how often participants responded to our prompts without a limit on how long they took to respond. In other words, if a participant received a prompt in the morning but waited until the next day to complete our RQA, we still counted this as a response. We calculated the response rate in this way because it is well documented that people respond to emails and SMS text messages at their convenience rather than at the moment of reception [47]. As in study 2, we asked participants to rate their stress using an 11-point scale before and after the activity, and we report the change in this rating. We also report the time it took for participants to complete the RQA and the word count of their responses as proxies for engagement. We analyzed the interview responses using the same procedure that was applied to study 1; however, we did so with a new blank codebook.

### Ethical Considerations

We recognize that conducting research on mental health can raise several ethical issues; for example, our particular set of questions can induce stress or symptoms of depression and anxiety, particularly when participants are asked to recall a troubling situation. To mitigate these negative outcomes, we clearly explained the potential risks in the consent materials and reminded participants that the RQA was part of a research study. We also provided survey participants with the contact information of several mental health helplines. The interviewers were trained to clearly explain the goal of the project and maintain an appropriate level of empathy and support. Interviewers were also trained to run the Columbia-Suicide Severity Rating Scale protocol [48] if interviewees showed any indication of self-harm or suicidal ideation. Furthermore, interviewees had the option to skip any question they did not want to answer or to leave the interview session at any point. All our research activities were approved by the University of Toronto Research Ethics Board (36582).

## Results

### Study 1: User Perspectives After Onetime Use of Our RQA

During our first study, we elicited 4 major themes related to the benefits and pitfalls of our RQA for first-time users. We provide evidence for each theme in the following subsections.

#### Appreciation for Structured Reflection

Participants were appreciative of the fact that our RQA helped them break down the components of their stressful situation. By deconstructing the situation, participants felt that they were able to become more aware of the causes of their negative emotions, putting their thoughts “in the right order” (L2). Some of the participants also noted that the activity helped them recognize faulty thought patterns:

The activity helped me pinpoint my maladaptive coping strategy...[it] led me to think more with my brain and less with my immediate emotional reaction.

M17

#### Venting Negative Thoughts Through Writing

Participants enjoyed expressing their thoughts and feelings through writing because it allowed them to “get out all thoughts and feelings and take that weight off of my shoulders” (L5). Moreover, some of the participants appreciated seeing their thoughts typed out in front of them, commenting that the act of writing helped solidify previously nebulous or disjointed thoughts; for example, L4 thought that the RQA forced them to dissect their feelings that would have otherwise been unorganized.

M11 suggested that writing about their thoughts allowed them to examine their situation “from an outside perspective,” almost as if they were analyzing someone else’s situation instead of their own. This affordance made it easier for them to ignore personal tendencies and instead think more objectively about their thought process.

#### Helping Identify Solutions

Participants also stated that the activity prompted them to adopt a problem-solving approach to improve their situation. They could better identify the root cause of their stress because they were prompted to describe their troubling situation in a structured order, which made it easier for them to find a solution to their problem. As the final question of our RQA prompted users to consider alternative ways of thinking, participants such as L3 felt empowered because they were often able to emerge from the activity with at least 1 prototype solution.

#### Incidental Negative Side Effects

Our RQA did not unilaterally help people become less worried about their troubling situation. L5 noted that as they were considering an alternative line of thinking, they found it confusing to keep track of both their original thought process and the reframed one. L6 felt that this confusion led directly to frustration, whereas others were frustrated because they could not identify a solution by the end of the activity:

The questions just made me think about how much pain I was in and really didn’t offer any solution whatsoever to the stress.

M19

Some of the participants also felt at a loss when asked to think of alternative perspectives on their thoughts.

### Study 2: Comparing the RQA With a Baseline

#### Overview

Of the 215 participants, 111 (51.6%) were randomly assigned to the baseline condition and 104 (48.4%) to the RQA condition. The summary statistics for the measures that were collected during study 2 are shown in Table 2.

**Table 2:** Summary measures and statistics from study 2. Statistical significance between measures in the reflective questioning activity (RQA) and baseline conditions is indicated in the first column according to independent samples 1-tailed Welch t tests. Means are given with the SE within each condition. For each measure, we set our hypotheses as follows: the null hypothesis (H0) was that the mean for the RQA condition would be less than, or equal to, the mean for the baseline condition; by contrast, the alternative hypothesis (Ha) was that the mean for the RQA condition would be greater than the mean for the baseline condition.

Measure	RQA condition, mean (SD; SE)	Baseline condition, mean (SD; SE)	*t* test (*df*)	*P* value	Cohen *d*
Perceived utility^a^	1.2 (2.04; 0.2)	0.5 (2.11; 0.2)	2.82 (213)	.003	0.38
Perceived stress change^b^	0.7 (2.04; 0.2)	−0.4 (1.05; 0.1)	4.46 (213)	<.001	0.61
Completion time^b^ (min)	8.9 (8.16; 0.8)	1.6 (3.16; 0.3)	9.09 (213)	<.001	1.27
Response length^b^ (words)	87 (118.297; 11.6)	29 (57.95; 5.5)	4.52 (213)	<.001	0.63
Perceived time commitment	−0.2 (2.04; 0.2)	−0.3 (2.11; 0.2)	0.33 (213)	.37	0.05

^a^*P*<.05.

^b^*P*<.001.

#### Hypothesis 1 (Perceived Benefits)

Participants in the RQA condition saw significantly more utility in completing the activity than those in the baseline condition (*t*_213_=2.82; *P*=.003; Cohen *d*=0.38). The average rating for our RQA was 1.2 (SE 0.2), whereas the average rating for the baseline activity was 0.5 (SE 0.2). Although both these averages were near the neutral score of 0, there were many more positive ratings for our RQA. Of the 215 participants who used our RQA, n (79%) gave a nonneutral positive score, whereas only n (57%) did the same for the single-question activity. Participants also reported a statistically significant change in stress rating in the RQA condition compared with the baseline condition (*t*_213_=4.46; *P*<.001; Cohen *d*=0.61). Whereas people who used our RQA experienced a mean decrease of 0.7 (SE 0.2) point in their perceived stress rating, people who used the single-question activity actually experienced a mean increase of 0.4 point. A paired 1-tailed *t* test analyzing scores before and after engaging with the RQA condition indicated a statistically significant decrease in stress scores post-RQA, relative to their levels before starting it (*t*_103_=3.59; *P*<.001; Cohen *d*=0.36).

These results suggest that the additional questions from our RQA may not only help in potentially mitigating stress but also possibly counteract an initial increase in stress from revisiting the troubling situation.

#### Hypothesis 2 (Elapsed Time)

Participants in the RQA condition took 8.9 (SE 0.8) minutes on average to complete the activity, whereas those in the baseline condition took only 1.6 (SE 0.3) minutes on average; the difference between the 2 conditions according to this measure was statistically significant (*t*_213_=9.09; *P*<.001; Cohen *d*=1.27). We also found that participants wrote significantly longer responses while going through our RQA. Participants in the RQA condition wrote 87 (SE 11.6) words on average, whereas those in the baseline condition wrote 29 (SE 5.5) words on average; this difference was also statistically significant (*t*_213_=4.52; *P*<.001; Cohen *d*=0.63). Although the RQA required significantly more effort, there was no statistically significant difference in people’s subjective perceived time commitment (*t*_213_=0.33; *P*=.37; Cohen *d*=0.05). We conclude from these results that people found value in the additional time they spent completing the series of questions.

### Study 3: Observing Repeated Engagement With Our RQA

#### Overview

**Figure 1:**
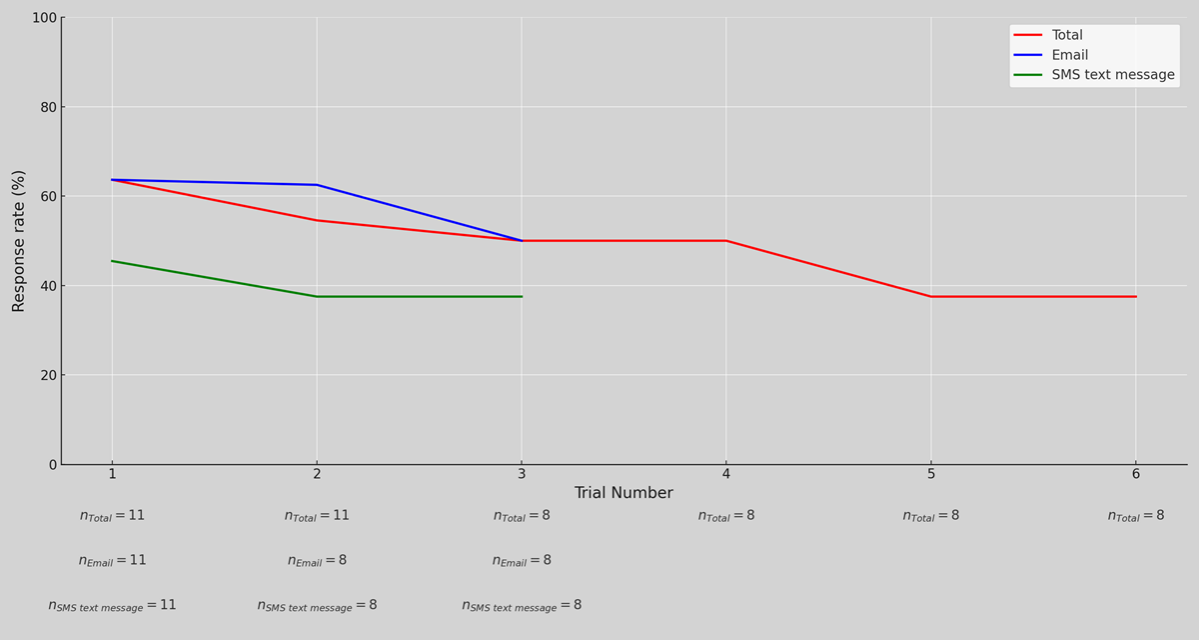
Participants’ response rate to our reflective questioning activity over the study period. The sample size for each data point is also shown below the respective trial number.

Figure 1 illustrates participants’ response rate to our RQA sent via email and SMS text message over the course of the study period. The figure not only shows the aggregated data across all interactions with our RQA but also splits the results according to the CMC platform through which the prompts were sent. We do not rely on quantitative data to claim that one way of delivering an RQA is better than the other; instead, we look into qualitative data to understand the role that technology plays in supporting long-term engagement with RQA.

#### Overall Engagement

We observed moderate engagement throughout the 2-week period of our study. We sent participants 54 prompts via email (n=27, 50%) and SMS text message (n=27, 50%), and participants completed the RQA in 27 (50%) of these cases. On 3 (11%) of these 27 occasions, participants completed the RQA twice in response to the same prompt; therefore, our RQA was actually completed 30 times during the study.

On average, people spent 18.5 (SE 1.2) minutes, wrote a total of 212 (SE 24.2) words, and experienced a stress level reduction of 1.2 (SE 0.3) points after completing the RQA.

Participants were much more engaged with our RQAs in this study compared with the AMT crowdworkers in study 2 (ie, they spent more time and typed longer responses), even as they completed it multiple times. One explanation for this discrepancy could be the amount of time participants were willing to commit to the study. Participants in study 2 likely completed our RQA in the midst of other crowdsourcing tasks or during their busy workdays. By contrast, participants from study 3 were able to pick a suitable time at their convenience, which in turn gave room for a longer time investment. A participant validated this hypothesis from their experiences:

Although I initially said that I would be available in the morning, I found the best time to do it in the time between 9 and 11 PM. I used to see the emails and text messages shortly after they came, but I used to only do them at my convenient times in the night.

D3

Our quantitative and qualitative data show that people could spend as little or as much time as they wanted with the activity without the need for significant scaffolding. In the interviews, participants expressed similar opinions about the benefits of our RQA as they did in our previous studies. Most notably, participants saw benefits to having a structured way of organizing thoughts because it helped them identify triggers and devise an alternate way of thinking.

#### Repeated Engagement With the RQA

A major goal of this study was to observe how participants engaged with the RQA over time. Unsurprisingly, we observed that the response rate decreased over time. Figure 1 shows that the response rate was 64% (7/11) when participants received their first prompt and then 55% (6/11) for the second prompt. By the time they had seen 6 prompts, the response rate went all the way down to 38% (3/8). When we asked participants to explain this trend during our interviews, the main complaint was that doing the same activity in such a short interval was boring and tedious. D3 mentioned that the length of the activity was acceptable for a onetime event, but when they had to do the activity thrice in the same week, it “came across as a chore.” Another participant expressed similar sentiments:

When it started coming every other day, I felt like I had to do a school homework. So I felt a little bit of pressure to do the activity.

D10

Participants expressed that they would have preferred to have larger intervals (eg, once a week) between the times they were requested to go through the RQA. This was not only because of the monotony of the task but also because participants struggled to think of new troubling situations to reflect upon:

By the time I got the last prompt, I could not find a stressful situation in my life. Maybe the frequency should vary depending on the amount of stress a person is going through.

D6

Participants acknowledged that regularly prompting them to do our activity had value. A few of them noted that they would have forgotten to revisit the RQA had they not been given periodic reminders. D1 also believed that they “got more comfortable with the activity [over time] and started setting aside a time to do the activity.”

Repeated engagement with our RQA also helped people form habits that yielded benefits outside of the activity itself; for example, D10 informed us that they did the activity multiple times in their mind either to think about how their previous responses could be improved or how they could apply these questions in a new situation. D4 found that doing the activity multiple times was a good mental exercise to prepare themselves for less stressful situations that they may encounter during the day.

#### CMC Platforms

Another goal of this study was to gain insights into the role of technology in deploying RQAs. Figure 1 shows that there was a noticeable difference between the response rates for email versus SMS text message. Even in our exit survey, 8 (73%) of the 11 participants said that they would prefer email over SMS text message for doing this activity, whereas the rest of the participants (3/11, 27%) had no preference. One of the main reasons for the preference was the affordances of desktop and laptop computers when it came to completing the RQA. Most notably, participants commented on how computers are better suited for reading and writing longer passages of text:

Typing is very difficult in mobile phones. The screen size is small and editing stuff is a nightmare. On the other hand, if you want to write a long answer, you would probably do that on the computer because the process is just much easier.

D1

Participants also felt that doing the RQA on a computer minimizes the chance for distractions; for example, D7 commented that sitting in front of their computer gave them the “right mindset to do the activity.” With a computer, they felt that they had control over their workspace because they could easily close other tabs and applications. By contrast, when they tried to do the activity on their mobile phone, there were cases when a call or a push notification disrupted their train of thought.

Although email was generally preferred for completing the RQA, many people agreed that mobile phones are a great mechanism for sending notifications and reminders. Some of the participants (eg, D4 and D6) expressed the concern that people may not check their emails as frequently as they check SMS text messages:

Most of the time, I have my phone in my hand, whereas I check my emails at most once or twice a day. So if you need me to do something immediately, you would probably need me to reach via text messages. I can respond to an email even 2 days later.

D4

Participants also informed us of instances when they switched between the 2 CMC platforms. When D6 was prompted to do the RQA over SMS text message, they sent the link to themselves over social media and then accessed it on their desktop computer to complete the RQA. Some of the participants posited that the 2 CMC platforms could be integrated into the same system:

What you can do is you can ask me to answer the questions in the text message, but at the same time you will also send me an email that has the links to the actual page.

D6

Alternatively, others suggested that the RQA could be advertised over social media platforms such as Facebook or Instagram because people normally access their accounts across multiple devices. In doing so, people could have the option to choose whichever platform they see fit.

## Discussion

### Principal Findings

In this work, we aimed to understand the benefits of a brief digital intervention that people could complete on their mobile phone or computer to lessen their concerns about a troubling situation. Our second study showed that doing the RQA could be more effective in reducing instantaneous stress compared with simply reflecting on a troubling situation without structured questions, whereas our first and third studies elicited qualitative findings that we hope will inform the design of future interventions in this space. Most notably, we found that participants appreciated the RQA for its ability to help them undergo a structured analysis of their troubling situation, identify solutions to improve their situation, and vent their negative feelings. Although participants felt that the series of questions was worth the additional time commitment, we also saw some obstacles toward long-term engagement with the RQA: the monotony of doing the same activity several times, the limited affordances of mobile phones, and the importance of having the prompts align with the occurrence of new troubling situations.

Our findings indicate that people from the general population saw value in engaging with a simple lightweight reflection activity without an active conversational partner. Although there has been significant research effort toward making mental health platforms more sophisticated and humanlike [37,49], our work shows that simpler interfaces can also yield benefits. Across all our studies, participants expressed that the structured nature of the RQA played a pivotal role in making them more aware of their troubling emotions. By deconstructing past events, participants were able to view their feelings in an organized manner and from a third-person perspective, enabling them to reevaluate whether their feelings were justified. The writing activity acted as a medium through which they could externalize repressed emotions, a helpful practice that has been noted by past psychology research [50]. People often falsely assume that their problems are a reflection of their own identity or their relationship with others. Failing to *separate problems from persons* can cause people to identify themselves as different from what society considers *normal*, eventually leading them to fixate on their negative traits [51]. Our RQA provided people with the opportunity to explore the relationship between their problem and their own self but from a different perspective.

The RQA also offered a general structure that people could adapt to their own life situations. We saw that a few of the participants applied the same line of questioning outside of the activity itself, hinting at longer-lasting benefits. We foresee that RQAs could serve as a gateway for people struggling with stress and depression to engage with more complex activities and therapeutic tools. Validating this potential would require a longer study, but our research already demonstrates the hurdles that RQA interventions must overcome to support long-term engagement.

### Improving the Design of RQAs

The success of the RQA in our work does not mean that future RQAs could not be even better. Although we observed an average decrease in participants’ stress levels after completing the RQA, some of the participants from study 1 remarked that the activity left them confused and without a concrete solution. We hypothesize that such concerns could be remedied by providing users with sample responses to each question as a source of inspiration. These examples could be curated by researchers, or they could be collected from previous users who voluntarily contributed their responses to a database [52]. Topic modeling could be used to tag the examples with keywords related to their subject matter, and an information retrieval system could rank the relevance of these examples [53]. Collaborative filtering could even be used to gradually collect ratings for each example and then tailor examples to individuals’ preferences [54].

Another way that RQAs could be made better is by personalizing the questions. The activity could ask users to rate the perceived benefit of each question, or we could use the average response length as a proxy for estimating the utility of each question. Using this information, we could extend or emphasize questions that individuals find most beneficial. We could also use this information to remove questions that induce stress. However, thought records and behavioral chaining are intentionally designed processes with many critical steps; therefore, removing questions may detract from the activity’s benefits.

Our 9-question RQA took inspiration from CBT principles, but future work could investigate RQA designs based on other psychological principles; for example, encouraging expressions of gratitude or social connections with others can play a key role in stress and depression management [55,56], and RQAs built around these practices can similarly help people manage their well-being. Future work could also explore different activity structures. Many of the participants (8/11, 73%) in study 3 complained about the inconvenience of typing on their smartphones; therefore, an alternative activity could ask people to record and listen to their own voices for reflection. Another activity could encourage peer support by starting conversations among online peers. Finally, researchers could create brief activities centered around other psychological frameworks beyond CBT, with past examples being centered around mindfulness [57], motivational interviews [58], and acceptance and commitment therapy [59].

### Considerations for Long-Term Engagement

Our 2-week deployment in study 3 enabled us to gain insights into how people would engage with RQAs over a period of time. Although participants were pleased with the fact that they could specify their hours of availability, receiving prompts for the RQA 3 times within the same week was overwhelming for most of them (3/8, 38%). The biggest criticism was that people received multiple prompts without experiencing a new troubling event; therefore, they either had to go through our RQA while analyzing the same event as before or recalling a troubling event from the distant past. Ideally, the frequency of prompts would adapt dynamically according to a person’s needs. A participant suggested that users should have control over how often they receive reminders to complete our RQA, explaining that individuals who experience more stress than others might benefit more from doing these activities in short intervals. Going a step further, future work could integrate physical activity trackers, smartphone sensors, and Internet of Things devices to automatically detect periods of heightened stress [60,61], turning our RQA into a just-in-time adaptive intervention.

Another issue with completing our RQA too often was that answering the same set of questions became boring and tedious; yet, adjusting the prompt frequency alone may not be enough to resolve these concerns. One way to add variety would be to mix an RQA with other microinterventions, as was done by Paredes et al [13] in their *PopTherapy* work. Brief interventions such as our RQA could also serve as a gateway to more time-consuming exercises or professional therapy. By giving people a preview of the potential improvement in the mood that they can receive from articulating their thoughts and emotions, habits can be formed, and users may become more motivated to build on this momentum [62].

### Limitations

Rather than developing a mental health intervention for people experiencing clinical depression or other psychological disorders, our intention was to design our RQA for as broad a population as possible. It would be imperative for researchers to conduct further studies specifically with individuals with mental health disorders to understand the benefits and potential risks of digitally delivered RQAs. We suspect that self-reflection could not only serve as a convenient mechanism for people to practice what they learn in psychotherapy but also perpetuate negative thought patterns. We also recognize that our participant cohorts—AMT crowdworkers and university students—do not represent all aspects of the general public. Most of our qualitative findings were not tied to participants’ specific contexts, and we did not find any obvious evidence of substantial differences among the cohorts. Nevertheless, future work could deploy RQAs to more diverse populations.

### Conclusions

In this work, we used CBT principles to design a brief RQA that helps people articulate, reflect on, and change their thoughts and emotions about a troubling situation. The 3 studies we presented in our paper provide evidence that people are willing to engage with, and find value in, brief self-reflection activities delivered through CMC platforms, even without scaffolding such as training or real-time feedback. We found that providing people with a brief online activity not only helped them reduce their perceived stress levels related to a self-selected situation but also helped them challenge their potentially negative thought patterns and identify alternative ways of thinking. We also found that people were willing to use the RQA more than once, although future work is needed to strike a balance between utility and monotony. We hope that our work inspires other researchers to explore new formats for brief interventions that help people with their everyday struggles.

## Abbreviations

AMT: Amazon Mechanical Turk
CBT: cognitive behavioral therapy
CMC: computer-mediated communication
RQA: reflective questioning activity

## Data Availability

Anonymized data sets will be made available from the corresponding author upon reasonable request.
